# ﻿One new genus and two new species of the spider family Phrurolithidae (Arachnida, Araneae) from Xishuangbanna Tropical Botanical Garden, Southwest China

**DOI:** 10.3897/zookeys.1117.89211

**Published:** 2022-08-11

**Authors:** Keke Liu, Yuanhao Ying, Shuqiang Li

**Affiliations:** 1 College of Life Science, Jinggangshan University, Ji’an 343009, Jiangxi, China Jinggangshan University Ji’an China; 2 Institute of Zoology, Chinese Academy of Sciences, Beijing 100101, China Institute of Zoology, Chinese Academy of sciences Beijing China

**Keywords:** Eurasia, phrurolithid, taxonomy, types, Yunnan

## Abstract

*Edelithus***gen. nov.** is described based on the discovery and description of two new species from Xishuangbanna, Yunnan Province, China: *E.puer***sp. nov.** and *E.shenmiguo***sp. nov.** Both species are described in detail and illustrated. Types are deposited in the Institute of Zoology, Chinese Academy of Sciences (IZCAS) in Beijing, China.

## ﻿Introduction

The spider family Phrurolithidae Banks, 1892 includes 20 genera and 313 species from America, Australia, and Eurasia (WSC 2022). In China, 173 phrurolithid species are known belonging to 12 genera (Liu et al. 2022): *Abdosetae* Fu, Zhang & MacDermott, 2010, *Acrolithus* Liu & Li, 2022, *Aculithus* Liu & Li, 2022, *Alboculus* Liu, 2020, *Bosselaerius* Zamani & Marusik, 2020, *Corealithus* Kamura, 2021, *Grandilithus* Liu & Li, 2022, *Otacilia* Thorell, 1897, *Pennalithus* Kamura, 2021, *Phrurolithus* C.L. Koch, 1839, *Phrurotimpus* Chamberlin & Ivie, 1935, and *Plynnon* Deeleman-Reinhold, 2001. It is clear that China has the most species- and genus-rich phrurolithid fauna (Wang et al. 2020; Li et al. 2021; Yao et al. 2021; Hong et al. 2022; Zhu et al 2022).

While studying the phrurolithid species from Xishuangbanna Tropical Botanical Garden in Yunnan Province, China (XTBG; Li 2020), a new genus and two new species are found. The goal of this paper is to describe the new genus and species from XTBG.

## ﻿Materials and methods

Specimens were examined using a SZ6100 stereomicroscope. Both male and female copulatory organs were dissected and examined in 80% ethanol using an Olympus CX43 compound microscope with a KUY NICE CCD camera. The epigynes were cleared with pancreatin solution. Specimens, including dissected male palps and epigynes, were preserved in 75% ethanol after examination. For SEM photographs, the specimens were kept under natural dry conditions, coated with gold with a small ion-sputtering apparatus ETD-2000, and photographed with a Zeiss EVO LS15 scanning electron microscope. Types are deposited in the Institute of Zoology, Chinese Academy of Sciences (IZCAS) in Beijing, China.

The measurements were taken using a stereomicroscope (Axio Vision SE64 rel. 4.8.3) and are given in millimetres. The body lengths of all specimens exclude the chelicerae and spinnerets. Terminology of the male and female genitalia follows Ramírez (2014) and Liu et al. (2022).

Leg measurements are given as total length (femur, patella, tibia, metatarsus, tarsus). Promarginal and retromarginal teeth on the chelicerae are given as the fproximal, median and distal and counted from the base of the fang to the distal groove. Leg spines are documented by dividing each leg segment into four aspects: dorsal (d), prolateral (p), and retrolateral (r), and indicating the ventral (v) spines as single (1) or paired (2), e.g., femur I d2, pv1111; tibia d1, I v2222. The abbreviations used in the figures are as follows:

**Eyes**

**ALE** anterior lateral eye;

**AME** anterior median eye;

**MOA** median ocular area;

**PLE** posterior lateral eye;

**PME** posterior median eye.

**Legs**

**CS** chemsensory seta;

**CTC** claw tuft clasper;

**LO** lyriform organ;

**MPB** metatarsal preening brush;

**MTS** metatarsal dorsal stopper;

**Sc** scale;

**SS** slit sensillum;

**TS** tenent setae.

**Chelicerae**

**PES** promarginal escort seta;

**PRS** promarginal rake seta;

**RES** retromarginal escort seta;

**SS** slit sensillum;

**WS** whisker seta.

**Male palp**

**dTA** distal tegular apophysis;

**DTA** dorsal tibial apophysis;

**Em** embolus;

**FA** femoral apophysis;

**rTA** retrolateral tegular apophysis;

**RTA** retrolateral tibial apophysis;

**SD** sperm duct;

**sTA** subdistal tegular apophysis;

**VTA** ventral tibial apophysis.

**Epigyne**

**Bu** bursa;

**CD** copulatory duct;

**CO** copulatory opening;

**CT** connecting tube;

**FD** fertilization duct;

**GA** glandular appendage;

**MS** median septum;

**Spe** spermathecae.

## ﻿Taxonomy

### ﻿Family Phrurolithidae Banks, 1892

#### ﻿ Edelithus

Taxon classificationAnimaliaAraneaePhrurolithidae

Liu & Li gen. nov.

7CE30421-DCC1-5A2F-93C3-2F8ACA55333F

https://zoobank.org/59555B23-B0D4-4DD9-B5C9-3BCF0E6C98EE

##### Type species.

*Edelithusshenmiguo* Liu & Li sp. nov. by designation herein.

##### Diagnosis.

**Figure 1.**
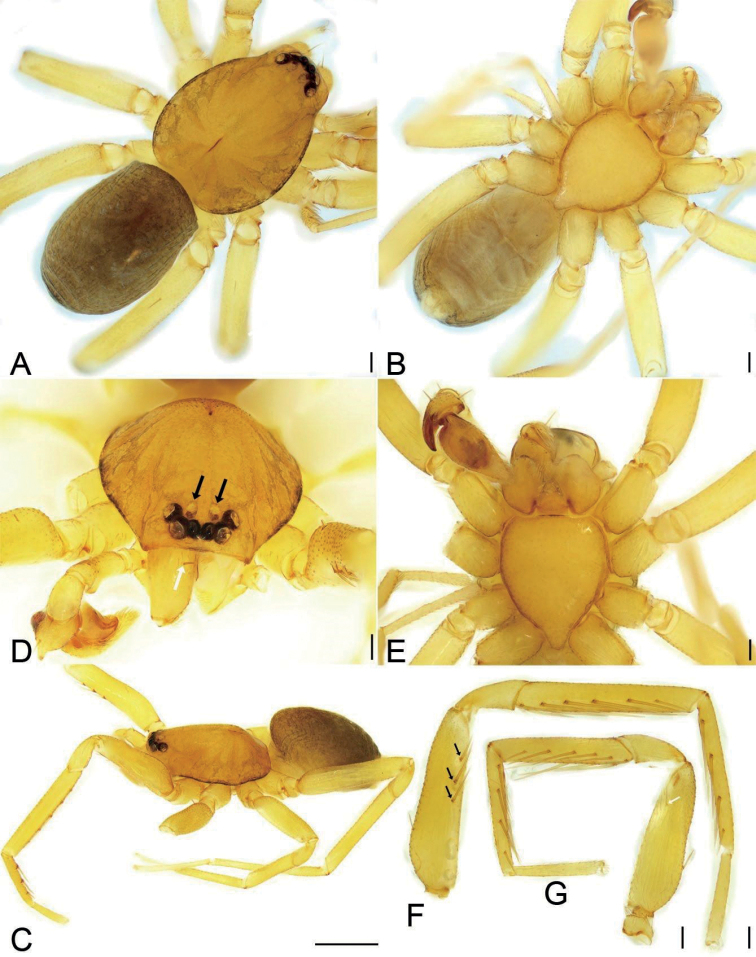
*Edelithusshenmiguo* sp. nov., male **A** habitus, dorsal view **B** same, ventral view **C** same, lateral view **D** carapace, dorsal view, white arrow to cheliceral spine, black arrow to oval posterior median eyes without black annulations **E** same, ventral view **F** leg I, prolateral view, black arrows to prolateral spines on femur **G** leg II, white arrow to prolateral spine on femur. Scale bars: 0.1 mm (**A, B, D–G**), 0.5 mm (**C**).

**Figure 2.**
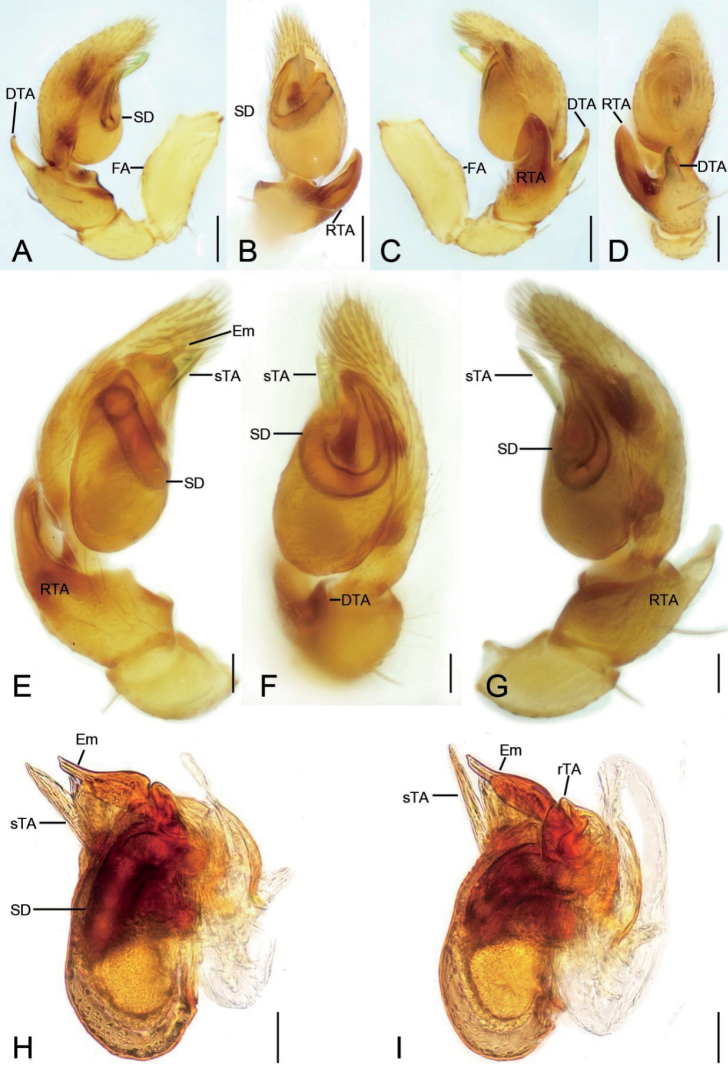
*Edelithusshenmiguo* sp. nov., male palps **A** holotype, prolateral view **B** same, ventral view **C** same, retrolateral view **D** same, dorsal view **E** paratype, prolateral view **F** same, ventral view **G** same, retrolateral view **H, I** tegulum, retrolateral view. Abbreviations: DTA – dorsal tibial apophysis, Em – embolus, FA – femoral apophysis, rTA – retrolateral tegular apophysis, RTA – retrolateral tibial apophysis, SD – sperm duct, sTA – subdistal tegular apophysis. Scale bars: 0.1 mm.

**Figure 3.**
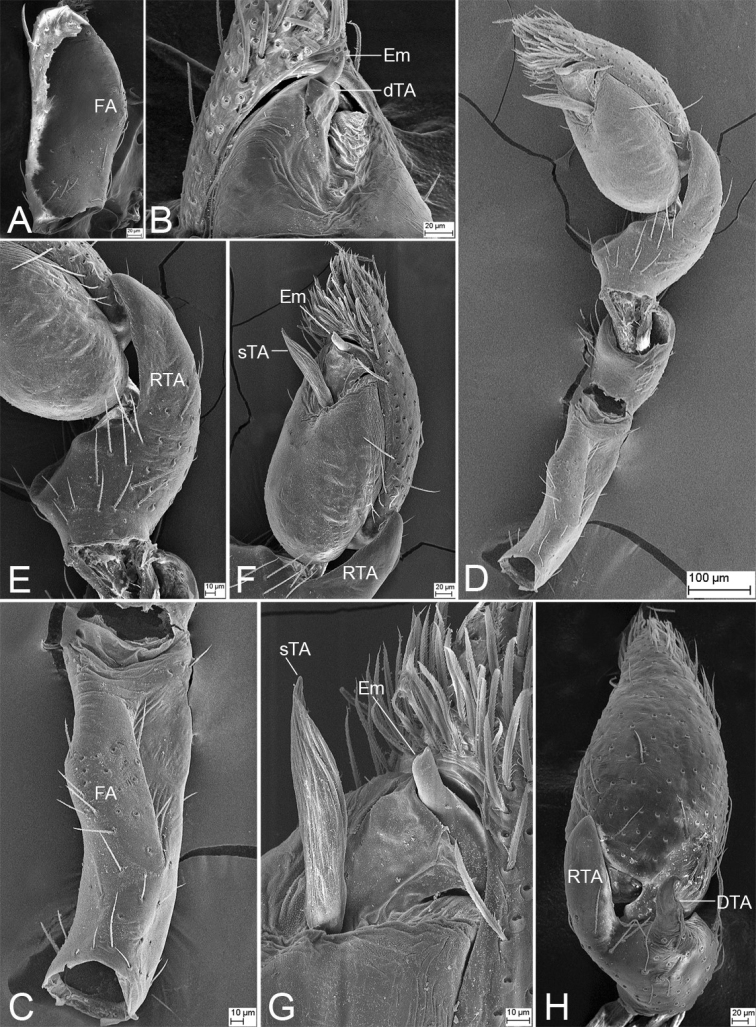
SEM micrographs of *Edelithusshenmiguo* sp. nov., male palp **A** femur, prolateral view **B** ventral view, detail of tegular end **C** femur, ventral view **D** retrolateral view **E** retrolateral view, detail of retrolateral tibial apophysis **F** retrolateral view, detail of tegulum **G** same, detail of tegular end **H** same, detail of tibial apophyses. Abbreviations: dTA – distal tegular apophysis, DTA – dorsal tibial apophysis, Em – embolus, FA – femoral apophysis, RTA – retrolateral tibial apophysis, SD – sperm duct, sTA – subdistal tegular apophysis.

**Figure 4.**
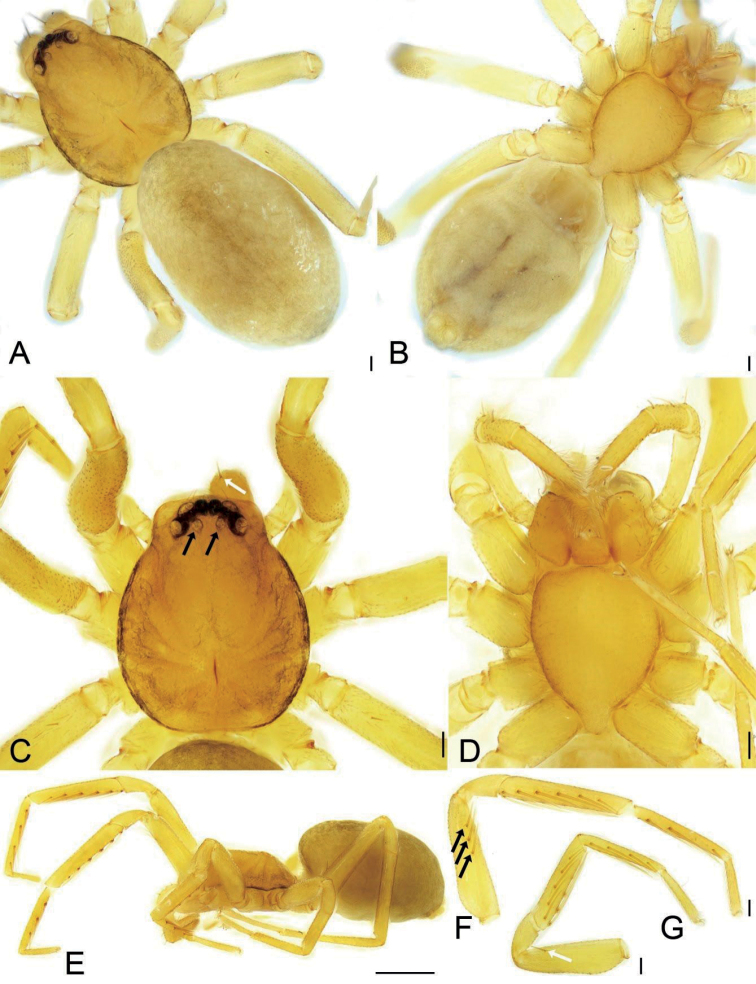
*Edelithusshenmiguo* sp. nov., female **A** habitus, dorsal view **B** same, ventral view **C** same, lateral view **D** carapace, dorsal view, white arrow to cheliceral spine, black arrow to oval posterior median eyes without black annulations **E** same, ventral view **F** leg I, prolateral view, white arrows to prolateral spines on femur **G** Leg II, white arrow to prolateral spine on femur. Scale bars: 0.1 mm (**A, B, D–G**); 0.5 mm (**C**).

**Figure 5.**
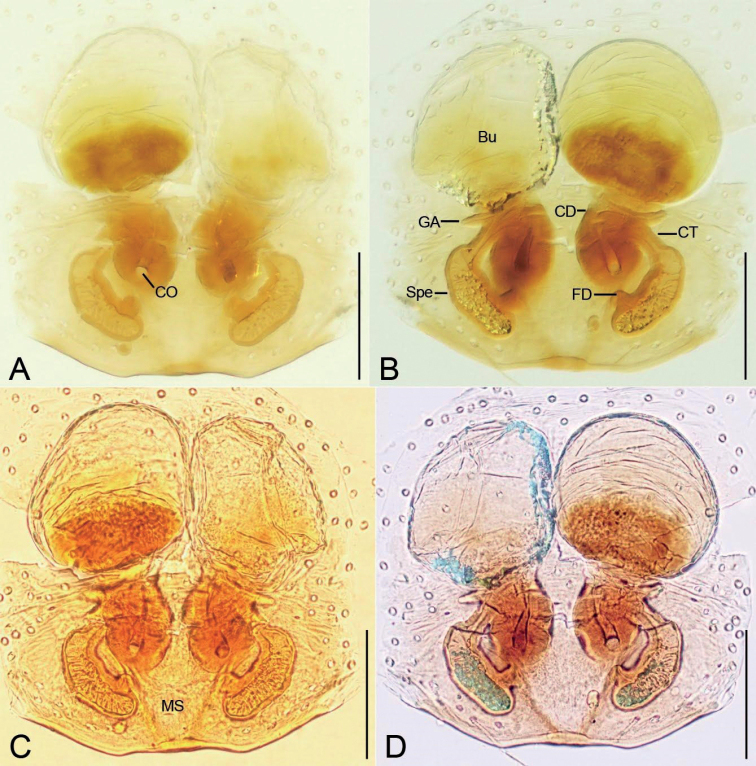
*Edelithusshenmiguo* sp. nov., female **A** epigyne, ventral view **B** same, dorsal view **C** same, ventral view **D** same, dorsal view. Abbreviations: Bu – bursa, CD – copulatory duct, CO – copulatory opening, CT – connecting tube, FD – fertilization duct, GA – glandular appendage, MS – median septum, Spe – spermatheca. Scale bars: 0.1 mm.

**Figure 6.**
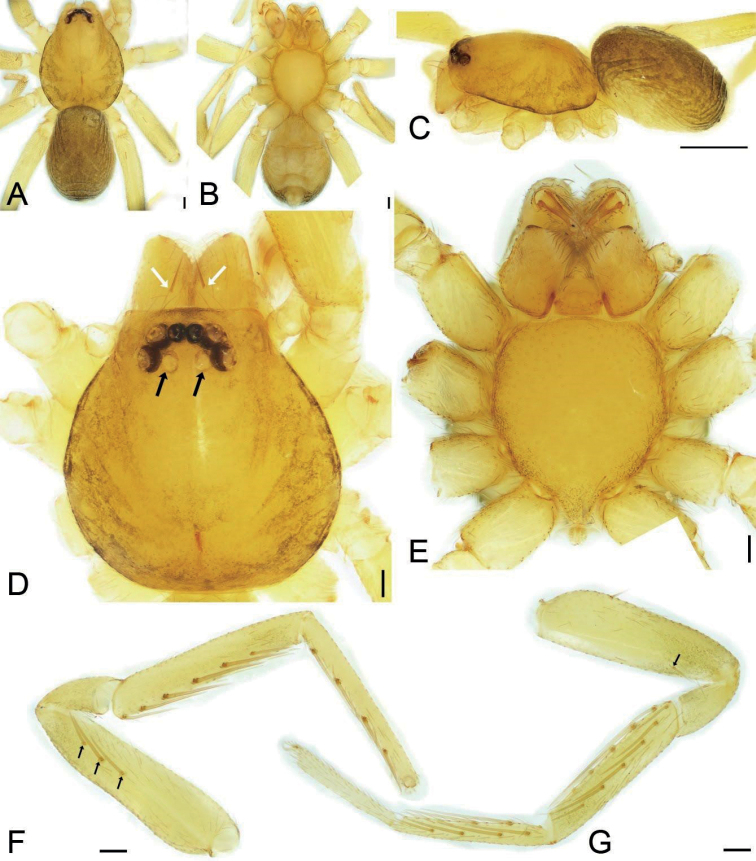
*Edelithuspuer* sp. nov., male **A** habitus, dorsal view **B** same, ventral view **C** same, lateral view **D** carapace, dorsal view, white arrows to cheliceral spines, black arrow to oval posterior median eyes without black annulations **E** endites, labium and sternum, ventral view **F** left leg I, prolateral view, black arrows to prolateral spines on femur **G** left leg II, black arrow to prolateral spine on femur. Scale bars: 0.1 mm (**A, B, D–G**); 0.5 mm (**C**).

The new genus differs from *Labialithus* Kamura, 2021 (see Kamura 2021: figs 9F–J, 10B, C) by the small PME with indistinct black pigment around the eye cup (vs large PME with clear pigment around the eye cup in *Labialithus*) (Figs 1D, 4C, 6D, 11D), the femora I with one dorsal spine (vs absent in *Labialithus*) and three prolateral spines (vs one in *Labialithus*) (Figs 1F, 4F, 6F, 8A, 11F) and the metatarsi III–IV lacking ventral spines (vs usually with two pairs in *Labialithus*), the male scutum covering nearly 1/2 of abdomen (vs more than 2/3 in *Labialithus*) and by the palpal tibia with a dorsal apophysis (vs absent in *Labialithus*). It can be separated from *Otacilia* (see Wang et al. 2015: fig. 14A; Liu et al. 2022: suppl. 2, figs 72, 74, 75, 77–79, 81, 82, 84, 85, 87, 88, 90, 91, 93–96, 98, 99, 101–105, 107–109, 111, 113, 114, 116–118, 120, 124, 137, 141) by the light abdomen lacking dark stripes (vs present in *Otacilia*) (Figs 1A, 4A, 6A, 11A), femora II lacking prolateral spine (in most specimens) or with one prolateral spine (in the few specimens) (vs 2–4 spines in *Otacilia*) (Figs 1G, 4G, 6G, 8C, 11G), the palpal femur with a weakly protruded ventral apophysis (vs moderately or strongly protruded in *Otacilia*) (Figs 2, 3, 9, 10) and the small, short embolus (vs relatively large hook-shaped or spine-like embolus) (Figs 2, 3, 9, 10). Male of this genus can be easily distinguished from *Phrurolithus* (see Wang et al. 2015: fig. 15C–E; Zamani and Marusik 2020: figs 4A–C, E, F, 7A–E) by the scutum covering nearly 1/2 of abdomen (vs nearly entire abdomen in *Phrurolithus*) (Figs 1A, 6A) and by the palpal tibia with a dorsal apophysis (vs absent *Phrurolithus*) (Figs 2D, 3H, 9E, 10I). Females of this genus can be separated from the genus *Labialithus* by the very small, widely separated copulatory openings without atrium (vs relatively large, slightly separated copulatory openings with distinct atrium) (Figs 5, 12). Furthermore, *Edelithus* spp. differ from some phrurolithid genera by the tarsal claws lacking tooth (Fig. 8B, D, I), while present in *Acrolithus* and *Aculithus* Liu & Li, 2022 with three teeth, in *Alboculus* with two teeth, and in *Grandilithus* and *Otacilia* with four teeth (see Liu et al. 2020a: fig. 5J; Liu et al. 2022: figs 4C, D, G, H, L, P, 38D, E, H, K, O, 122B, C, E, I, M), but in *Phrurolithus* only with degenerated and inconspicuous blunt teeth (Ramírez 2014: fig. 75E).

##### Etymology.

The name is a combination of the first three letters of “*edentatus*” (referring to the tarsal claws lacking tooth) and the latter half of *Phrurolithus*. The gender is masculine.

##### Description.

**Figure 7.**
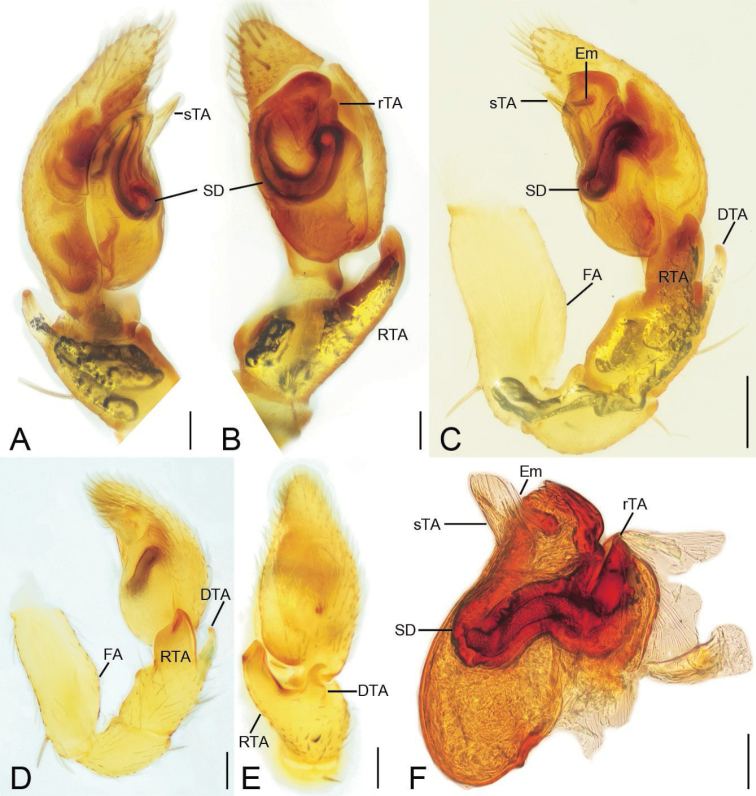
*Edelithuspuer* sp. nov., male palps **A** holotype, prolateral view **B** same, ventral view **C, D** same, retrolateral view **E** same, dorsal view **F** tegulum of paratype, retrolateral view. Abbreviations: DTA – dorsal tibial apophysis, Em – embolus, FA – femoral apophysis, rTA – retrolateral tegular apophysis, RTA – retrolateral tibial apophysis, SD – sperm duct, sTA – subdistal tegular apophysis. Scale bars: 0.1 mm.

**Figure 8.**
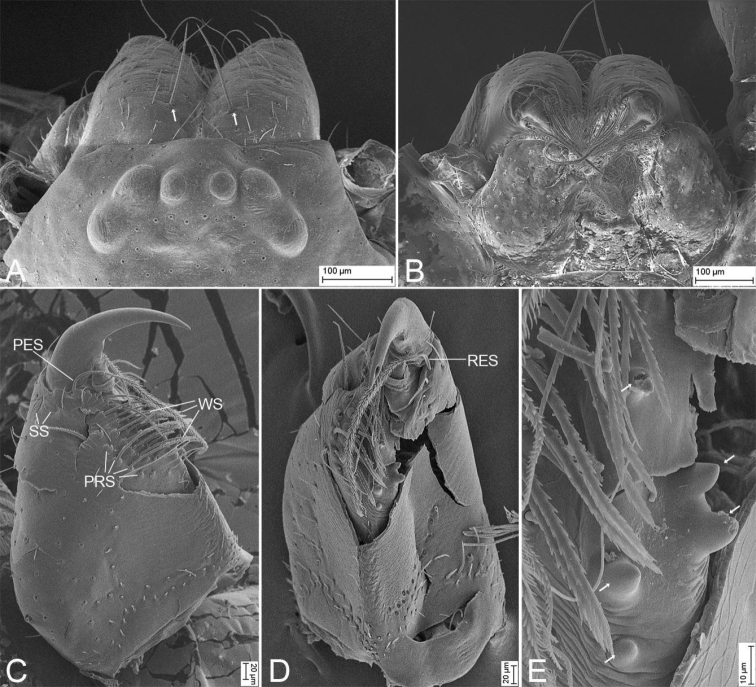
SEM micrographs of *Edelithuspuer* sp. nov., male **A** eyes and chelicerae, dorsal view, white arrows to cheliceral spines **B** chelicerae, endites, and labium, ventral view **C** chelicera, prolateral view **D** same, ventral view **E** same, ventral view, close-up, white arrows to details of teeth. Abbreviations: PES – promarginal escort seta, PRS – promarginal rake setae, RES – retromarginal escort seta, SS – slit sensillum, WS – whisker setae.

Small, body length 1.0–2.5. Eyes (Figs 1D, 4C, 6D, 7A, 11D): AER straight and PER procurved in dorsal view, AME clearly smaller than other eyes, PME with indistinct black pigment around eye cups, smaller than ALE and PLE, nearly separated by their diameter. Chelicera (Figs 1D, 4A, 6D, 7A, B, 11D) with one frontal strong spine, three promarginal and two retromarginal teeth. Legs without annulations and stripes. Femora I–IV with one dorsal spine each (Figs 1F, G, 4F, G, 6F, G, 8A, C, 11F, G), femur I with three prolateral spines, and femur II with one prolateral spine or none, tibiae I and II with six pairs of ventral spines; metatarsi I and II with tour pairs of ventral spines. Scutum (Figs 1A, 6A) covers nearly 1/2 of abdomen in males, but absent in females (Figs 4A, 11A).

**Figure 9.**
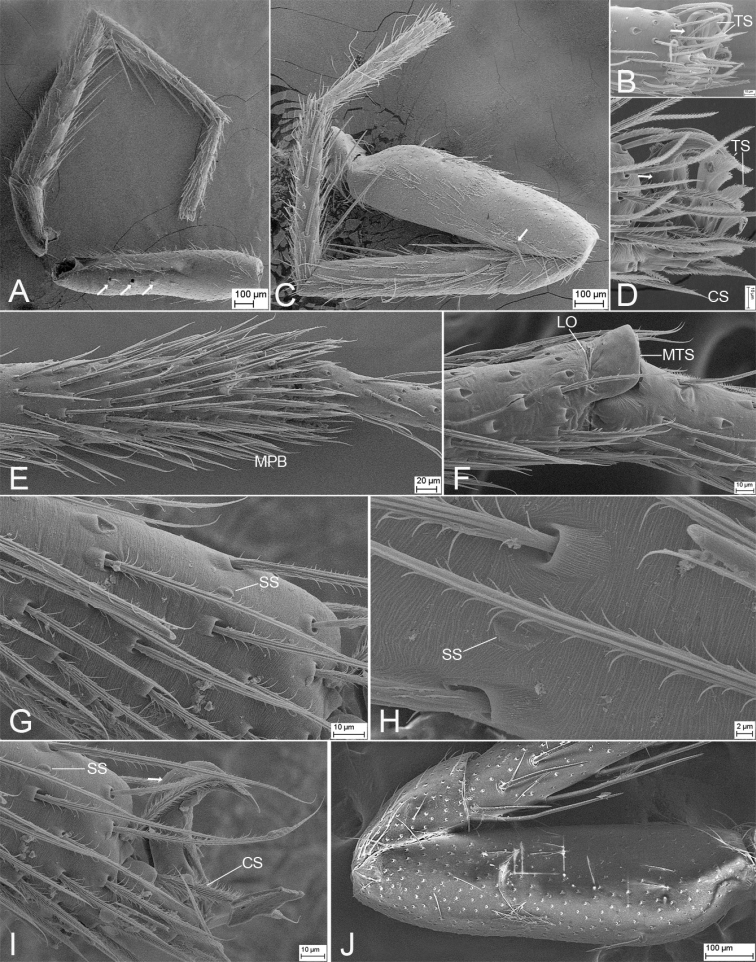
SEM micrographs of *Edelithuspuer* sp. nov., male **A** left leg I, white arrows to detail of prolateral spines, prolateral view **B** same, tarsal claws, prolateral view **C** left leg II, white arrow to detail of prolateral spine, prolateral view **D** same, detail of claw tuft setae **E** left leg III, detail of metatarsal preening brush, prolateral view **F** left leg IV, metatarsus-tarsus joint, prolateral view **G** same, detail of tarsal end, prolateral view **H** same, tarsus, detail of the tarsal organ, prolateral view, slightly dorsal **I** same, tarsal claw and claw tuft setae, prolateral view **J** left femur II, prolateral view. Abbreviations: CS – chemosensory seta, LO – lyriform organ, MPB – metatarsal preening brush, MTS – metatarsal dorsal stopper, SS – slit sensillum, TS – tenent setae.

**Figure 10.**
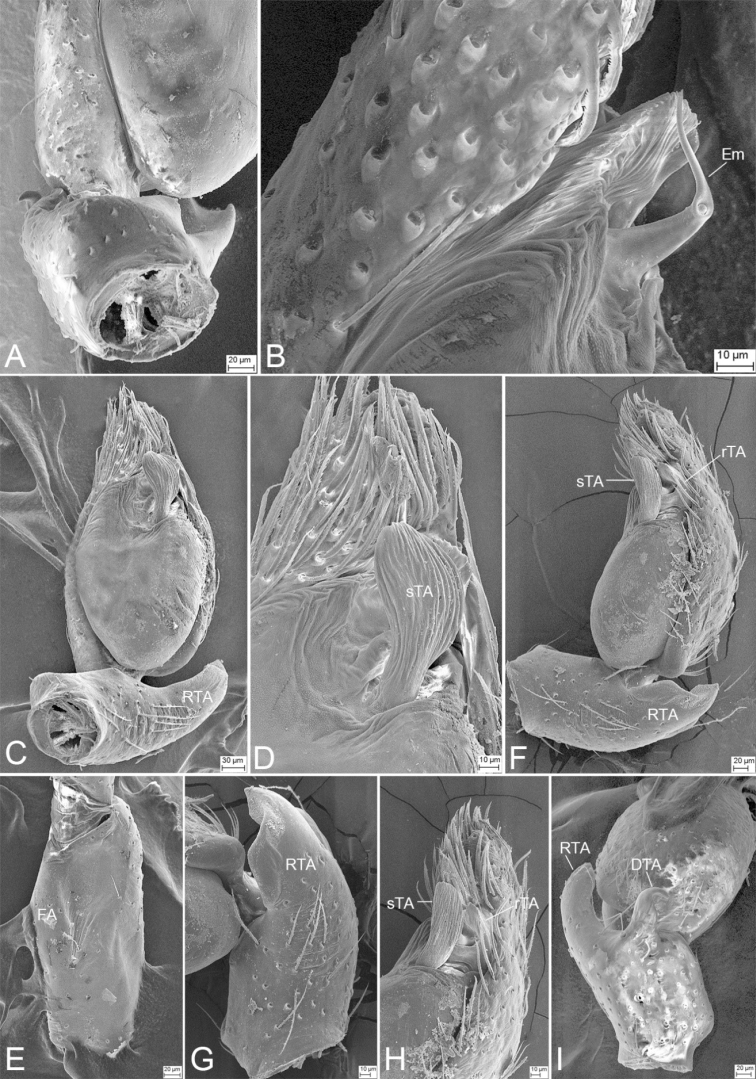
SEM micrographs of *Edelithuspuer* sp. nov., male palp **A** prolateral view, slightly ventral **B** same, detail of embolus **C** ventral view **D** same, detail of subdistal tegular apophysis **E** femur, retrolateral view **F** retrolateral view **G** same, detail of retrolateral tibial apophysis **H** same, detail of tegular end **I** dorsal view. Abbreviations: DTA – dorsal tibial apophysis, Em – embolus, FA – femoral apophysis, rTA – retrolateral tegular apophysis, RTA – retrolateral tibial apophysis, sTA – subdistal tegular apophysis.

***Male palp*** (Figs 2, 3, 9, 10): femur with a weak ventral extension; tibia with two well-developed apophyses, retrolateral apophysis very thick, as long as or shorter than tibia, dorsal apophysis hook-shaped, shorter than the retrolateral one; tegulum with a leaf-shaped subdistal apophysis and a blunt retrolateral apophysis; embolus short, shorter than subdistal tegular apophysis, with a round sperm pore, touching subdistal tegular apophysis.

***Epigyne*** (Figs 5, 12) with a pair of small copulatory openings, located posteriorly or subposteriorly; median septum absent or located posteriorly; bursae large, covering nearly 1/2 of epigynal plate, anteriorly located.

##### Composition.

*Edelithuspuer* sp. nov. and *E.shenmiguo* sp. nov.

##### Distribution.

China (Yunnan Province).

#### ﻿ Edelithus puer

Taxon classificationAnimaliaAraneaePhrurolithidae

Liu & Li sp. nov.

FA7670D1-A2AE-5DB0-A1D4-03511C9F1D19

https://zoobank.org/73417E12-1129-408E-9A01-046FC40865FF

Figs 1 , 2 , 3 , 4 , 5

##### Material examined.

***Holotype*** ♂ (Phu-147), 21°54.607'N, 101°17.005'E, elevation ca 633 m, XTBG, Menglun Township, Mengla County, Xishuangbanna, Yunnan Province, China, 4–11.IV.2007, G. Zheng leg. ***Paratypes*** 1 ♂, 2 ♀, the same data as holotype; 1 ♀, 4–11.IV.2007, other data as holotype (JSIII-2-18); 1 ♀, 10–20.VI.2007, other data as holotype (JSIII-1-20); 1 ♀, 1–15.VIII.2007, other data as holotype (JSIII-3-23); 3 ♂, 16–31.III.2007, other data as holotype (JSIII-5-16); 1 ♀, 10–20.VI.2007, other data as holotype (JSIII-2-20); 1 ♀, 16–31.V.2007, other data as holotype (JSIII-1-20); 2 ♂, 1–15.IV.2007, other data as holotype (JSIII-5-17); 5 ♂, 1 ♀, 1–15.IV.2007, other data as holotype (JSIII-2-17); 2 ♂, 1–15.IV.2007, other data as holotype (JSIII-4-17); 3 ♂, 2 juveniles, 1–15.IV.2007, other data as holotype (JSIII-3-17); 1 ♀, 19–26.V.2007, other data as holotype (JSIII-2-17); 1 ♀, 16–31.VI.2007, other data as holotype (JSIII-5-22); 3 ♀, 16–31.IV.2007, other data as holotype (JSIII-5-18); 2 ♀, 4–11.V.2007, other data as holotype (JSIII-3-18); 1 ♀, 4–11.V.2007, other data as holotype (JSIII-1-19); 1 ♀, 19–26.V.2007, other data as holotype (JSIII-2-17); 3 ♀, 16–31.IV.2007, other data as holotype (JSIII-3-22); 1 ♀, 4–11.V.2007, other data as holotype (JSIII-1-18); 1 ♀, 19–26.IV.2007, other data as holotype (JSIII-3-17); 1 ♀, 1–15.V.2007, other data as holotype (JSIII-5-19); 1 ♀, 10–20.VI.2007, other data as holotype (JSIII-3-20); 1 ♂, 16–31.IV.2007, other data as holotype (JSIII-4-18); 1 ♀, 16–31.V.2007, other data as holotype (JSIII-3-20); 1 ♀, 19–26.IV.2007, other data as holotype (JSIII-4-17); 2 ♀, 19–26.V.2007, other data as holotype (JSIII-2-19); 6 ♂, 1 ♀, 16–31.IV.2007, other data as holotype (JSIII-1-18); 1 ♀, 19–26.V.2007, other data as holotype (JSIII-4-19); 6 ♂, 1 ♀, 16–31.III.2007, other data as holotype (JSIII-1-16); 3 ♂, 16–31.III.2007, other data as holotype (JSIII-1-16); 2 ♂, 16–31.III.2007, other data as holotype (JSIII-3-16); 1 ♀, 1–15.V.2007, other data as holotype (JSIII-2-19); 1 ♀, 19–25.XI.2007, other data as holotype (JSIII-3-03); 4 ♂, 2 ♀, 1–15.IV.2007, other data as holotype (JSIII-1-17); 2 ♂, 1–15.III.2007, other data as holotype (JSIII-3-15); 1 ♂, 16–31.IV.2007, other data as holotype (JSIII-3-18); 1 ♂, 1 ♀, 16–31.IV.2007, other data as holotype (JSIII-2-18); 2 ♀, 16–31.VI.2007, 21°55.428'N, 101°16.441'E, elevation ca 598 m, other data as holotype (CZI-3-22); 1 ♀, 16–31.VI.2007, other data as holotype (CZI-5-22); 1 ♀, 16–31.VI.2007, other data as holotype (CZI-2-22); 4 ♂, 16–31.VI.2007, 21°54.984'N, 101°16.982'E, elevation ca 656 m, other data as holotype (JSIII-5-18); 1 ♀, 4–11.V.2007, other data as previous (JSII-3-18); 1 ♀, 10–20.VI.2007, other data as previous (JSII-2-20); 1 ♀, 16–31.VI.2007, other data as previous (JSIII-4-18); 2 ♂, 1–15.III.2007, other data as previous (JSII-5-15); 1 ♀, 1–15.V.2007, other data as previous (JSII-2-19); 3 ♀, 4–11.IV.2007, other data as previous (JSII-2-16); 5 ♂, 19–26.III.2007, other data as previous (JSII-4-15); 7 ♂, 1–15.IV.2007, other data as previous (JSII-2-17); 2 ♀, 1–15.V.2007, other data as previous (JSII-5-19); 4 ♂, 16–31.III.2007, other data as previous (JSII-4-16); 2 ♂, 1–15.III.2007, other data as previous (JSII-1-15); 2 ♀, 19–26.V.2007, other data as previous (JSII-4-19); 2 ♂, 16–31.III.2007, other data as previous (JSII-5-16); 2 ♂, 1–15.IV.2007, other data as previous (JSII-4-17); 6 ♂, 2 ♀, 16–31.IV.2007, other data as previous (JSII-3-18); 3 ♂, 1–15.IV.2007, other data as previous (JSII-1-17); 2 ♀, 4–11.V.2007, other data as previous (JSII-2-18); 1 ♀, 16–31.IV.2007, other data as previous (JSII-5-22); 3 ♀, 4–11.V.2007, other data as previous (JSII-4-18); 1 ♀, 19–26.V.2007, other data as previous (JSII-1-19); 2 ♀, 1–15.V.2007, other data as previous (JSII-1-19); 2 ♀, 16–31.IV.2007, other data as previous (JSII-4-22); 6 ♂, 16–31.III.2007, other data as previous (JSII-1-16); 2 ♂, 1 ♀, 16–31.IV.2007, other data as previous (JSII-2-18); 4 ♂, 16–31.III.2007, other data as previous (JSII-3-16); 6 ♂, 1–15.IV.2007, other data as previous (JSII-3-17); 3 ♀, 19–26.IV.2007, other data as previous (JSII-4-17); 2 ♀, 4–16.IV.2007, other data as previous (JSII-4-16); 1 ♀, 10–20.VI.2007, other data as previous (JSII-4-20); 1 ♀, 16–31.V.2007, other data as previous (JSII-3-20); 3 ♂, 1 ♀, 1–15.IV.2007, other data as previous (JSII-5-17); 1 ♀, 16–31.V.2007, other data as previous (JSII-5-20); 1 ♀, 19–26.IV.2007, other data as previous (JSII-1-17); 1 ♀, 19–26.IV.2007, other data as previous (JSII-2-17); 5 ♂, 16–31.III.2007, other data as previous (JSII-2-16); 3 ♀, 1–15.VI.2007, other data as previous (JSII-5-21); 1 ♀, 1–15.VII.2007, other data as previous (JSII-5-23); 2 ♀, 1–15.VI.2007, other data as previous (JSII-3-21); 1 ♀, 1–15.VI.2007, other data as previous (JSII-2-21); 2 ♀, 1–15.VII.2007, other data as previous (JSII-2-23); 1 ♂, 16–31.III.2007, 21°54.718'N, 101°16.940'E, elevation ca 645 m, other data as holotype (JSI-4-16); 1 ♀, 19–26.IV.2007, other data as previous (JSI-3-17); 2 ♂, 1–15.III.2007, other data as previous (JSI-3-15); 1 ♂, 16–31.IV.2007, other data as previous (JSI-5-18); 4 ♂, 1–15.IV.2007, other data as previous (JSI-4-17); 4 ♀, 16–31. VII.2007, other data as previous (JSI-2-24); 2 ♂, 10–20.VI.2007, other data as previous (JSI-3-20); 2 ♀, 1–15.V.2007, other data as previous (JSI-2-19); 1 ♀, 1–15.IV.2007, other data as previous (JSI-4-21); 1 ♀, 10–20.IV.2007, other data as previous (JSI-1-20); 2 ♀, 1–15.VI.2007, other data as previous (JSI-2-21); 2 ♀, 1–15.VII.2007, other data as previous (JSI-2-23); 5 ♂, 16–31.III.2007, other data as previous (JSI-1-16); 1 ♂, 1–15.IV.2007, other data as previous (JSI-3-17); 2 ♀, 16–31.V.2007, other data as previous (JSI-5-20); 1 ♀, 16–24.X.2007, other data as previous (JSI-2-06); 3 ♂, 1 ♀, 16–31.V.2007, other data as previous (JSI-1-20); 1 ♀, 16–31.VII.2007, other data as previous (JSI-3-24); 1 ♀, 4–11.V.2007, other data as previous (JSI-2-18); 3 ♂, 1–15.IV.2007, other data as previous (JSI-5-17); 1 ♀, 16–31.VII.2007, other data as previous (JSI-5-24); 2 ♂, 2 ♀, 19–26.IV.2007, other data as previous (JSI-4-17); 1 ♀, 4–11.V.2007, other data as previous (JSI-3-18); 2 ♂, 1–15.III.2007, other data as previous (JSI-2-15); 1 ♂, 2 ♀, 16–31.V.2007, other data as previous (JSI-4-20); 1 ♀, 1–15.V.2007, other data as previous (JSI-5-19); 2 ♀, 4–11.IV.2007, other data as previous (JSI-1-16); 1 ♀, 19–26.IV.2007, other data as previous (JSI-2-17); 1 ♀, 19–26.V.2007, other data as previous (JSI-3-19); 1 ♂, 1 ♀, 16–31.V.2007, other data as previous (JSI-2-20); 1 ♀, 10–20.VI.2007, other data as previous (JSI-4-20); 1 ♀, 19–26.V.2007, other data as previous (JSI-2-19); 1 ♂, 1 ♀, 1–15.V.2007, other data as previous (JSI-1-19); 1 ♀, 4–11.IV.2007, other data as previous (JSI-2-16); 1 ♀, 10–20.VI.2007, other data as previous (JSI-2-20); 1 ♀, 16–31.IV.2007, other data as previous (JSI-4-18); 6 ♂, 1 ♀, 16–31.IV.2007, other data as previous (JSI-3-18); 5 ♂, 4 ♀, 16–31.IV.2007, other data as previous (JSI-2-18); 8 ♂, 16–31.III.2007, other data as previous (JSI-2-16); 4 ♂, 1–15.IV.2007, other data as previous (JSI-1-17); 5 ♂, 1 ♀, 16–31.III.2007, other data as previous (JSI-3-16); 10 ♂, 2 ♀, 1–15.IV.2007, other data as previous (JSI-2-17); 1 ♂, 16–31.V.2007, other data as previous (JSI-3-30); 1 ♀, 19–26.V.2007, other data as previous (JSI-4-19); 2 ♂, 2 ♀, 16–31.IV.2007, other data as previous (JSI-1-18).

##### Etymology.

The specific name refers to a famous tea from Xishuangbanna, Pu’er tea, which is planted on the mountainsides of Xishuangbanna and has a long history in China; noun in apposition.

##### Diagnosis.

The new species can be distinguished from *E.shenmiguo* sp. nov. (Figs 9, 10, 12) by the retrolateral tegular apophysis with bent apex (vs straight) and the very short embolus lacking spine-like tip (vs the relatively long embolus with a spine-like tip) in male palp (Figs 2, 3) and the triangular median septum (vs absent), the stout copulatory ducts (vs slender) and the C-shaped spermathecae (vs oval) in female epigyne (Fig. 5).

##### Description.

**Male** (holotype). Habitus as in Fig. 1A–C. Total length 1.95, carapace 0.99 long, 0.78 wide, abdomen 0.92 long, 0.65 wide. Eye sizes and interdistances (Fig. 1A, D): AME 0.04, ALE 0.06, PME 0.05, PLE 0.06; AME–AME 0.03, AME–ALE 0.01, PME–PME 0.04, PME–PLE 0.04, AME–PME 0.05, AME–PLE 0.09, ALE–ALE 0.13, PLE–PLE 0.21, ALE–PLE 0.03; PME separated by slightly less than their diameters. MOA 0.14 long, frontal width 0.11, posterior width 0.13. Chelicerae (Fig. 1B, D, E) with three promarginal (median largest, distal smallest) and two retromarginal teeth (distal larger). Endites (Fig. 1B, E) slightly oblique, brush shaped, anterolateral area of endite with row of thick serrula and six long, thick setae. Labium wider than long, anteriorly with 10–12 setae. Sternum (Fig. 1E), longer than wide, lateral margin thickened, with weak precoxal triangles and lacking intercoxal extensions, posteriorly triangular, blunt end. Legs (Fig. 1): measurements: I 3.29 (0.90, 0.35, 0.84, 0.76, 0.44); II 3.85 (0.73, 0.48, 0.97, 0.99, 0.68); III 2.53 (0.66, 0.32, 0.48, 0.60, 0.47); IV 3.74 (0.96, 0.37, 0.84, 0.95, 0.62); spination: femora I d1, pv111, II d1, III d1, IV d1; tibiae I v222222, II v222221, metatarsi I v2221, II v2221. Scutum (Fig. 1A) nearly covering 1/2 of abdomen.

***Colouration*** (Fig. 1A–C). Carapace yellow, with radial, irregular light yellow-brown stripes submarginally and arc-shaped dark stripes around margin. AME, ALE and PLE with dark layer of black pigment around the eye cup, but PME absent. Chelicerae, endites, and labium yellow. Sternum yellow, mottled around margin. Legs yellow, without dark stripes. Abdomen yellow-brown, mottled, with dark brown net-shaped stripes; venter yellow.

***Palp*** (Figs 2, 3). Femoral apophysis weak, with shallow groove and one strong dorsal spine near distal femur. Retrolateral tibial apophysis large, thick, finger-like, longer than tibia. Dorsal tibial apophysis longer than 1/2 length of retrolateral tibial apophysis, with broad base and a small hook-shaped tip, subdistal part with a strong constriction. Sperm duct V-shaped, reaching subposterior part of tegulum. Distal tegular apophysis lamellate, membranous, touching the base of embolus, covered by subdistal tegular apophysis in ventral view. Subdistal tegular apophysis gramineous leaf-shaped, membranous, slightly less than 1/2 of tegular length. Embolus very short, horn-like, less than 1/3 length of subdistal tegular apophysis, covered by subdistal tegular apophysis. Sperm opening round, located in subapical part.

**Female.** Habitus as in Fig. 4. Total length 2.21, carapace 0.92 long, 0.75 wide, abdomen 1.27 long, 0.83 wide. As in male, except as noted. Eye sizes and interdistances (Fig. 4A, D): AME 0.04, ALE 0.07, PME 0.04, PLE 0.06, AME–AME 0.02, AME–ALE 0.01, PME–PME 0.06, PME–PLE 0.04, AME–PME 0.04, AME–PLE 0.09, ALE–ALE 0.12, PLE–PLE 0.20, ALE–PLE 0.03. MOA 0.12 long, frontal width 0.10, posterior width 0.13. Leg (Fig. 4A, B) measurements: I 4.05 (1.07, 0.48, 1.04, 0.95, 0.51); II 2.61 (0.67, 0.35, 0.54, 0.60, 0.45); III 2.37 (0.63, 0.29, 0.45, 0.57, 0.43); IV 3.41 (0.89, 0.37, 0.74, 0.89, 1.060.52). Leg spination (Fig. 4): tibiae II v22222, metatarsi I v2222, II v2222.

***Colouration*** (Fig. 4A, B). Lighter than male.

***Epigyne*** (Fig. 5). Epigynal plate slightly longer than wide, subposterolaterally with pair of round copulatory openings, posteriorly with triangular median septum. Copulatory ducts short and thick, slghtly shorter than spermathecae. Bursae large round, touching, covering nearly 1/2 of epigynal plate. Glandular appendages short, transversal, directed laterally, less than the length of copulatory ducts. Connecting tubes very short, nearly as long as glandular appendages. Spermathecae nearly C-shaped, widely separated by median septum. Fertilization ducts short, located posteriorly on spermathecae, directed anterolaterally.

##### Comments.

The detailed study of a large number of these specimens revealed that most specimens (ca 9/10) lack prolateral spine on femora I, but a few specimens (ca 1/10) with one prolateral spine which locate at the distal part of femora I.

##### Distribution.

Known only from the type locality in Yunnan Province, China.

#### ﻿ Edelithus shenmiguo

Taxon classificationAnimaliaAraneaePhrurolithidae

Liu & Li sp. nov.

98D60652-0B6F-52BC-9A7B-159B69C2249F

https://zoobank.org/C4A6AD1-0DEE-4CCF-82A6-F14ABA2A6B66

**Figure 11.**
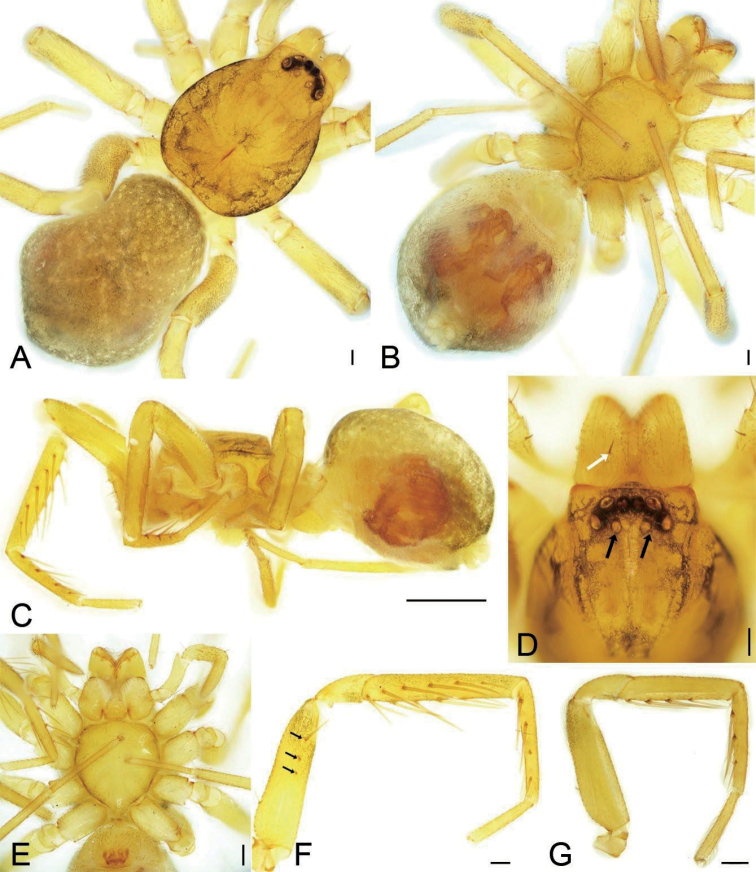
*Edelithuspuer* sp. nov., male **A** habitus, dorsal view **B** same, ventral view **C** same, lateral view **D** carapace, dorsal view, white arrow to cheliceral spine, black arrow to oval posterior median eyes without black annulations **E** same, ventral view **F** leg I, prolateral view, black arrows to prolateral spines on femur **G** leg II, prolateral view. Scale bars: 0.1 mm (**A, B, D–G**); 0.5 mm (**C**).

**Figure 12.**
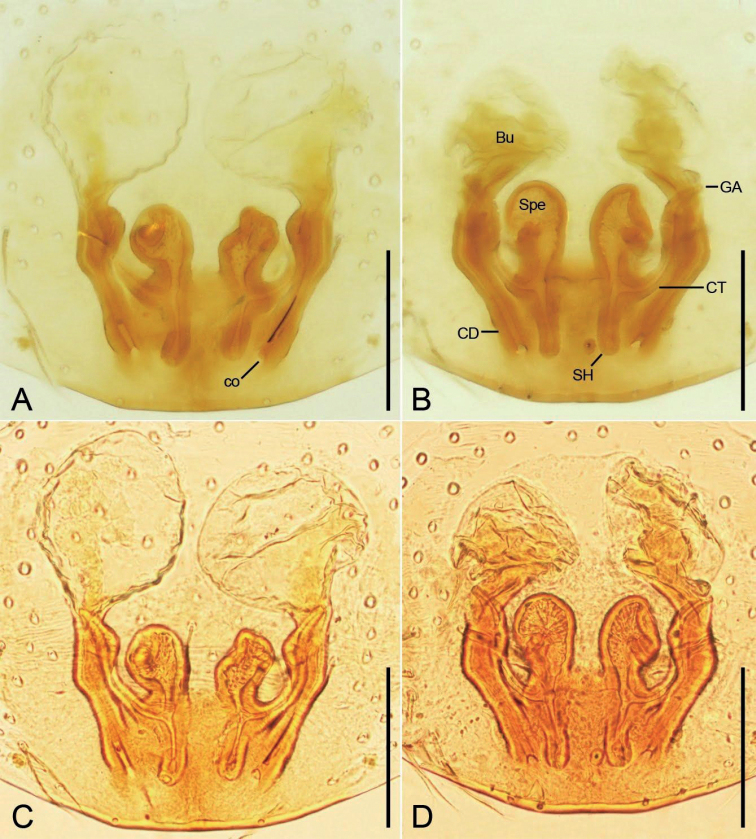
*Edelithuspuer* sp. nov., female. **A** epigyne, ventral view **B** same, dorsal view **C** same, ventral view **D** same, dorsal view. Abbreviations: Bu – bursa, CD – copulatory duct, CO – copulatory opening, CT – connecting tube, FD – fertilization duct, GA – glandular appendage, Spe – spermatheca. Scale bars: 0.1 mm.

Figs 6 , 7 , 8 , 9 , 10 , 11 , 12

##### Type material.

***Holotype*** ♂ (Phu-145, GBII-4-10), 21°57.669'N, 101°11.893'E, elevation ca 790 m, XTBG, Menglun Township, Mengla County, Xishuangbanna, Yunnan Province, China, 5–12.I.2007, G. Zheng leg. ***Paratype*** 2 ♂, 1 ♀, the same data as holotype (GBII-2-17); 11 ♂, 1 ♀, 16–31.III.2007, other data as holotype (GBII-1-16); 3 ♀, 4–11.IV.2007, other data as holotype (GBII-4-16); 5 ♂, 4 ♀, 16–31.III.2007, other data as holotype (GBII-2-16); 25 ♂, 3 ♀, 16–31.III.2007, other data as holotype (GBII-4-16); 2 ♂, 16–31.III.2007, other data as holotype (GBII-4-12); 2 ♂, 5–12.II.2007, other data as holotype (GBII-3-10); 1 ♀, 16–31.VII.2007, other data as holotype (GBII-4-24); 1 ♂, 2 ♀, 16–31.IV.2007, other data as holotype (GBII-4-18); 2 ♀, 1–15.V.2007, other data as holotype (GBII-1-19); 4 ♀, 10–20.VI.2007, other data as holotype (GBII-1-20); 3 ♀, 1–15.VII.2007, other data as holotype (GBII-3-23); 2 ♀, 19–26.IV.2007, other data as holotype (GBII-4-17); 3 ♂, 1 ♀, 5–12.I.2007, other data as holotype (GBII-2-10); 6 ♀, 4–11.V.2007, other data as holotype (GBII-3-18); 9 ♂, 3 ♀, 1–15.III.2007, other data as holotype (GBII-2-15); 1 ♂, 5–12.II.2007, other data as holotype (GBII-4-12); 2 ♂, 2 ♀, 19–26.III.2007, other data as holotype (GBII-3-15); 2 ♂, 5–12.I.2007, other data as holotype (GBII-2-12); 1 ♂, 1–15.I.2007, other data as holotype (GBII-2-11); 1 ♀, 10–20.VII.2007, other data as holotype (GBII-4-21); 3 ♀, 19–26.IV.2007, other data as holotype (GBII-2-17); 2 ♀, 1–15.I.2007, other data as holotype (GBII-1-23); 1 ♀, 10–20.VII.2007, other data as holotype (GBII-1-21); 3 ♂, 1 ♀, 1–15.III.2007, other data as holotype (GBII-5-15); 7 ♀, 19–26.V.2007, other data as holotype (GBII-1-19); 1 ♂, 19–26.V.2007, other data as holotype (GBII-3-19); 2 ♂, 1–15.II.2007, other data as holotype (GBII-4-13); 9 ♂, 1 ♀, 16–31.III.2007, other data as holotype (unspecified); 1 ♂, 16–31.III.2007, other data as holotype (GBII-2-20); 2 ♀, 5–12.III.2007, other data as holotype (GBII-4-14); 1 ♀, 1–15.V.2007, other data as holotype (GBII-4-19); 1 ♀, 1–15.IV.2007, other data as holotype (GBII-2-21); 1 ♀, 1–15.IV.2007, other data as holotype (GBII-1-21); 1 ♀, 5–12.XII.2007, other data as holotype (GBII-1-08); 2 ♀, 1–15.IV.2007, other data as holotype (GBII-4-21); 4 ♂, 1–15.III.2007, other data as holotype (GBII-2-13); 3 ♂, 1–15.III.2007, other data as holotype (GBII-3-15); 10 ♂, 2 ♀, 16–31.IV.2007, other data as holotype (GBII-1-18); 9 ♀, 4–11.V.2007, other data as holotype (GBII-1-18); 2 ♂, 1–15.IV.2007, other data as holotype (GBII-1-17); 2 ♀, 10–20.VI.2007, other data as holotype (GBII-4-20); 1 ♀, 10–14.VIII.2006, other data as holotype (GBII-4-01); 2 ♀, 19–26.IV.2007, other data as holotype (GBII-2-17); 7 ♂, 1 ♀, 16–31.III.2007, other data as holotype (GBII-5-16); 1 ♂, 1 ♀, 19–25.II.2007, other data as holotype (GBII-4-13); 4 ♂, 1 ♀, 1–15.IV.2007, other data as holotype (GBII-5-17); 7 ♂, 16–31.II.2007, other data as holotype (GBII-2-14); 2 ♂, 1 ♀, 1–15.IV.2007, other data as holotype (GBII-4-17); 1 ♂, 16–31.II.2007, other data as holotype (GBII-3-14); 1 ♀, 4–11.V.2007, other data as holotype (GBII-2-18); 1 ♀, 19–26.IV.2007, other data as holotype (GBII-3-17); 1 ♀, 4–11.IV.2007, other data as holotype (GBII-1-16); 5 ♀, 19–26.V.2007, other data as holotype (GBII-4-19); 5 ♀, 4–11.V.2007, other data as holotype (GBII-4-18); 1 ♀, 2–12.III.2007, other data as holotype (GBII-3-14); 1 ♀, 5–12.III.2007, other data as holotype (GBII-2-14); 2 ♂, 1 ♀, 19–25.I.2007, other data as holotype (GBII-2-11); 7 ♂, 16–31.III.2007, other data as holotype (GBII-3-16); 5 ♀, 19–26.V.2007, other data as holotype (GBII-2-19); 2 ♀, 10–20.VI.2007, other data as holotype (GBII-2-20); 1 ♀, 10–20.VI.2007, other data as holotype (GBII-3-20); 2 ♀, 16–31.IV.2007, other data as holotype (GBII-5-18); 4 ♀, 4–11.IV.2007, other data as holotype (GBII-2-16); 1 ♀, 16–31.IV.2007, other data as holotype (GBII-3-22); 1 ♂, 21°54.813'N, 101°12.634'E, elevation ca 876 m, 1–15.IV.2007, other data as holotype (GBII-4-17); 5 ♂, 1–15.IV.2007, other data as previous (GBIII-3-17); 1 ♂, 1–15.IV.2007, other data as previous (GBIII-5-17); 5 ♂, 1–15.IV.2007, other data as previous (GBIII-2-17); 1 ♀, 1–15.VII.2007, other data as previous (GBIII-2-23); 1 ♀, 19–26.IV.2007, other data as previous (GBIII-4-17); 4 ♂, 16–31.IV.2007, other data as previous (GBIII-3-18); 2 ♀, 4–11.V.2007, other data as previous (GBIII-3-18); 2 ♀, 19–26.IV.2007, other data as previous (GBIII-2-19); 1 ♂, 4–11.IV.2007, other data as previous (GBIII-1-16); 2 ♂, 1–15.III.2007, other data as previous (GBIII-1-15); 1 ♀, 16–31.V.2007, other data as previous (GBIII-3-20); 1 ♂, 16–31.IV.2007, other data as previous (GBIII-4-18); 2 ♀, 10–20.IV.2007, other data as previous (GBIII-4-20); 3 ♀, 19–26.V.2007, other data as previous (GBIII-3-19); 1 ♂, 1 ♀, 4–11.IV.2007, other data as previous (GBIII-4-16); 2 ♀, 16–26.V.2007, other data as previous (GBIII-4-19); 2 ♀, 4–11.V.2007, other data as previous (GBIII-4-18); 2 ♂, 1 ♀, 16–31.III.2007, other data as previous (GBIII-4-16); 4 ♂, 16–31.III.2007, other data as previous (GBIII-5-16); 1 ♀, 10–20.VII.2007, other data as previous (GBIII-4-18); 5 ♀, 21°57.445'N, 101°12.997'E, elevation ca 744 m, 4–11.V.2007, other data as holotype (GBIII-1-18); 1 ♀, 16–31.V.2007, other data as previous (GBIII-4-20); 3 ♀, 19–26.IV.2007, other data as previous (GBIII-3-17); 3 ♀, 19–26.III.2007, other data as previous (GBIII-4-15); 1 ♂, 6 ♀, 4–11.V.2007, other data as previous (GBIII-3-18); 4 ♀, 4–11.V.2007, other data as previous (GBIII-2-18); 2 ♀, 4–11.IV.2007, other data as previous (GBIII-3-16); 1 ♂, 5–12.II.2007, other data as previous (GBIII-4-12); 5 ♀, 4–11.V.2007, other data as previous (GBIII-4-18); 1 ♀, 16–31.V.2007, other data as previous (GBIII-1-20); 8 ♂, 1 ♀, 16–31.III.2007, other data as previous (GBIII-1-16); 2 ♀, 10–20.VI.2007, other data as previous (GBIII-1-20); 3 ♀, 16–31.VI.2007, other data as previous (GBIII-2-22); 1 ♀, 1–15.V.2007, other data as previous (GBIII-2-19); 6 ♀, 19–26.IV.2007, other data as previous (GBIII-4-17); 2 ♀, 1–15.V.2007, other data as previous (GBIII-4-19); 1 ♂, 1–15.III.2007, other data as previous (GBIII-5-15); 2 ♀, 19–26.IV.2007, other data as previous (GBIII-1-17); 8 ♀, 19–26.V.2007, other data as previous (GBIII-4-19); 1 ♂, 19–26.III.2007, other data as previous (GBIII-3-15); 7 ♂, 1 ♀, 16–31.III.2007, other data as previous (GBIII-3-16); 4 ♂, 1–15.IV.2007, other data as previous (GBIII-1-17); 1 ♂, 5–12.I.2007, other data as previous (GBIII-4-10); 1 ♀, 5–12.VI.2006, other data as previous (GBIII-1-06); 2 ♂, 19–26.III.2007, other data as previous (GBIII-2-15); 1 ♂, 16–31.III.2007, other data as previous (GBIII-3-05); 3 ♀, 4–11.IV.2007, other data as previous (GBIII-4-16); 1 ♀, 16–31.II.2007, other data as previous (GBIII-4-14); 6 ♂, 1–15.IV.2007, other data as previous (GBIII-2-17); 3 ♀, 19–26.V.2007, other data as previous (GBIII-1-19); 1 ♀, 16–31.V.2007, other data as previous (GBIII-3-20); 1 ♀, 1–15.IV.2007, other data as previous (GBIII-5-21); 1 ♀, 5–12.III.2007, other data as previous (GBIII-3-14); 1 ♂, 19–26.III.2007, other data as previous (GBIII-1-15); 1 ♀, 10–20.VI.2007, other data as previous (GBIII-3-20); 2 ♀, 10–20.VI.2007, other data as previous (GBIII-4-20); 13 ♂, 16–31.III.2007, other data as previous (GBIII-4-16); 7 ♂, 3 ♀, 1–15.IV.2007, other data as previous (GBIII-4-17); 7 ♂, 3 ♀, 16–31.III.2007, other data as previous (GBIII-2-16); 2 ♀, 4–11.IV.2007, other data as previous (GBIII-2-16); 1 ♀, 10–20.VI.2007, other data as previous (GBIII-3-21); 1 ♂, 5–12.III.2007, other data as previous (GBIII-2-14); 1 ♀, 16–24.IX.2006, other data as previous (GBIII-3-04); 2 ♂, 1–15.IV.2007, other data as previous (GBIII-5-17); 1 ♂, 1–15.IV.2007, other data as previous (GBIII-3-17); 3 ♂, 8 ♀, 5–12.III.2007, other data as previous (GBIII-4-14); 11 ♂, 10–31.III.2007, other data as previous (GBIII-5-16); 2 ♂, 5–12.III.2007, other data as previous (GBIII-1-14); 5 ♀, 16–29.VI.2007, other data as previous (GBIII-3-19); 2 ♀, 5–12.XI.2007, other data as previous (GBIII-2-06); 1 ♀, 16–31.IV.2006, other data as previous (GBIII-5-18); 2 ♀, 16–31.VI.2006, other data as previous (GBIII-4-22); 1 ♂, 4 ♀, 21°55.035'N, 101°16.500'E, elevation ca 558 m, 16–31.V.2007, other data as holotype (GZI-4-20); 1 ♀ (GBIII-4-12).

##### Etymology.

The specific name refers to the Chinese name of *Synsepalumdulcificum* (Schumach. & Thonn.) Daniell, 1852, shenmiguo, which was introduced to XTBG from Ghana; noun in apposition.

##### Diagnosis.

The new species can be distinguished from *E.puer* sp. nov. (Figs 2, 3, 5) by the ridge-shaped retrolateral tegular apophysis (vs bent) and the relatively long embolus with a spine-like tip (vs the very short embolus lacking spine-like tip) in male palp (Fig. 10B, H), and the epigynal plate lacking median septum (vs present), the relatively long, thin copulatory duct (vs very short and thick) and the oval spermathecae (vs C-shaped) (Fig. 12) in female epigyne (Fig. 12).

##### Description.

**Male** (holotype). Habitus as in Fig. 6A–C. Total length 1.93, carapace 1.03 long, 0.82 wide, abdomen 1.00 long, 0.70 wide. Eye sizes and interdistances (Fig. 6A, D): AME 0.04, ALE 0.06, PME 0.05, PLE 0.06, AME–AME 0.03, AME–ALE 0.01, PME–PME 0.05, PME–PLE 0.03, AME–PME 0.04, AME–PLE 0.09, ALE–ALE 0.12, PLE–PLE 0.22, ALE–PLE 0.03. MOA 0.13 long, frontal width 0.10, posterior width 0.14. Chelicerae (Fig. 7) with three promarginal (median largest, distal smallest) and two retromarginal teeth (distal larger); promarginal and retromarginal escort setae present, longer than fang; promarginal cheliceral whisker setae in a line; promarginal rake setae in three lines, comb-shaped; promarginal and retromarginal base of fang with two slit sensilla. Endites (Fig. 6E, 7B) slightly oblique, brush shaped, anterolateral area of endite with a row of thick serrula and a row of eight long and thick setae. Labium (Figs 6E, 7B) wider than long, anteriorly with 12 setae. Sternum (Fig. 6E), longer than wide, laterally with weak precoxal triangles and lacking intercoxal extensions, posteriorly triangular, blunt end. Leg measurements (Figs 6, 8): I 3.29 (0.93, 0.38, 0.88, 0.73, 0.42); II 3.85 (0.76, 0.34, 0.59, 0.62, 0.44); III 2.53 (0.64, 0.28, 0.48, 0.61, 0.42); IV 3.74 (0.98, 0.36, 0.83, 0.92, 0. 54). Leg spination (Figs 6, 8): femora I d1, pv111, II d1, III d1, IV d1; tibiae I v222222, II v222221; metatarsi I v2221, II v2221; metatarsi III and IV with conspicuous preening brushes, lyriform organs, and dorsal stoppers distally; tarsi with abundant scales, several long trichobothria dorsally, and several chemosensory setae on ventro-posterior tarsi and base of claws, slit sensillum located subdistally on dorsal part, oval, labium-shaped; inferior tarsal claw smooth without tooth, with a ventral scopula of tenent setae. Scutum (Fig. 6A) nearly covering 1/2 of abdomen.

***Colouration*** (Fig. 6A–E). Carapace yellow, with light yellow-brown spot in front of fovea, radial, irregular yellow-brown stripes submarginally and arc-shaped dark stripes around margin. AME, ALE and PLE with dark layer of black pigment around the eye cup, but PME absent. Chelicerae, endites, and labium yellow. Sternum yellow, mottled around margin. Legs yellow, without dark stripes. Abdomen yellow-brown, mottled, with three light yellow chevrons posteriorly and many yellow spots on surface; venter yellow.

***Palp*** (Figs 9, 10). Femoral apophysis weak, with shallow groove and one strong dorsal spine near distal femur. Retrolateral tibial apophysis large, thick, longer than tibia in retrolateral view, with blunt apex. Dorsal tibial apophysis shorter than retrolateral tibial apophysis, with a strong hook-shaped tip, submedial part with a strong constriction. Sperm duct U-shaped, reaching subposterior part of tegulum. Retrolateral tegular apophysis, arising from retrolateral tegulum, with two parts, one lamellate, transversely directed, touching the base of embolus, arising from retrolateral tegulum, the other ridge-like, anteriorly located in retrolateral view. Subdistal tegular apophysis fan-shaped, slightly less than 1/2 of tegular length. Embolus short, right-angled, with a spine-like tip, covered by subdistal tegular apophysis. Sperm pore round, located in the medial part of embolus, around the sharp turn, slightly less than the length of dorsal tibial apophysis.

**Female.** Habitus as in Fig. 11A–C. As in male, except as noted. Total length 2.20, carapace 0.99 long, 0.79 wide, abdomen 1.18 long, 0.92 wide. Eye sizes and interdistances (Fig. 11D): AME 0.04, ALE 0.06, PME 0.04, PLE 0.06, AME–AME 0.01, AME–ALE 0.01, PME–PME 0.05, PME–PLE 0.04, AME–PME 0.04, AME–PLE 0.08, ALE–ALE 0.12, PLE–PLE 0.20, ALE–PLE 0.02. MOA 0.13 long, frontal width 0.10, posterior width 0.13. Leg measurements (Fig. 11): I 2.99 (0.77, 0.36, 0.79, 0.67, 0.40); II 2.60 (0.72, 0.34, 0.57, 0.56, 0.41); III 2.39 (0.62, 0.30, 0.46, 0.56, 0.45); IV 3.50 (0.91, 0.34, 0.78, 0.88, 0.59). Leg spination (Fig. 11): femora II lacking prolateral spine; tibiae I v222221; metatarsi I v2222.

***Colouration*** (Fig. 11A–C). Lighter than male.

***Epigyne*** (Fig. 12). Epigynal plate longer than wide, posterolaterally with pair of slit-like copulatory openings. Copulatory ducts tube-shaped, longer than bursal diameter, submedially with a slight constriction. Bursae large oval, anteriorly located, slightly separated. Connecting tubes slender, less than length of copulatory ducts. Spermathecae oval, medially located, separated by half of their diameter. Spermathecal head parallel, posteromedially located, directed posteriorly, as long as spermathecal diameter, club-shaped. Fertilization ducts as long as spermathecal length, located at the center of spermathecae, directed laterally.

##### Comments.

Prolateral spine on femora I same detail as in *E.puer* sp. nov.

##### Distribution.

Known only from the type locality in Yunnan Province, China.

## Supplementary Material

XML Treatment for Edelithus

XML Treatment for Edelithus puer

XML Treatment for Edelithus shenmiguo
